# Overexpression of a soybean *YABBY* gene, *GmFILa*, causes leaf curling in *Arabidopsis thaliana*

**DOI:** 10.1186/s12870-019-1810-2

**Published:** 2019-06-03

**Authors:** Hui Yang, Guixia Shi, Xiao Li, Dezhou Hu, Yanmei Cui, Jinfeng Hou, Deyue Yu, Fang Huang

**Affiliations:** 1 National Center for Soybean Improvement, National Key Laboratory of Crop Genetics and Germplasm Enhancement, Jiangsu Collaborative Innovation Center for Modern Crop Production, Nanjing Agricultural University, Nanjing, 210095 China; 2 School of Life Sciences, Guangzhou University, Guangzhou, 510006 China; 3 Institute of Industrial Crops, Henan Academy of Agricultural Sciences, Zhengzhou, 450002 China

**Keywords:** Soybean, YABBY, *GmFILa*, Leaf, Adaxial-abaxial polarity, *Arabidopsis*

## Abstract

**Background:**

*YABBY* genes play important roles in the growth and polar establishment of lateral organs such as leaves and floral organs in angiosperms. However, the functions of *YABBY* homologous genes are largely unknown in soybean.

**Results:**

In this study, we identified *GmFILa* encoding a YABBY transcription factor belonging to *FIL* subfamily. In situ mRNA hybridization analysis indicated that *GmFILa* had specific expression patterns in leaf as well as in flower bud primordia. Ectopic expression of *GmFILa* in *Arabidopsis thaliana* altered the partial abaxialization of the adaxial epidermises of leaves. Besides, *GmFILa* transgenic plants also exhibited longer flowering period and inhibition of shoot apical meristem (SAM) development compared to the wild type plants. Digital expression data and quantitative real-time polymerase chain reaction (qRT-PCR) analysis demonstrated that the expression of *GmFILa* was induced by biotic and abiotic stresses and hormone treatments. Transcriptome analysis suggested that overexpressing *GmFILa* yielded 82 significant differentially expressed genes (DEGs) in *Arabidopsis* leaves, which can be classified into transcription factors, transporters, and genes involved in growth and development, metabolism, signal transduction, redox reaction and stress response.

**Conclusions:**

These results not only demonstrate the roles of *GmFILa* involved in leaf adaxial-abaxial polarity in *Arabidopsis*, but also help to reveal the molecular regulatory mechanism of *GmFILa* based on the transcriptomic data.

**Electronic supplementary material:**

The online version of this article (10.1186/s12870-019-1810-2) contains supplementary material, which is available to authorized users.

## Background

Several regulators controlling leaf abaxial-adaxial polarity and leaf growth have been identified in *Arabidopsis*, such as AS2 (ASYMMETRIC LEAVES2), class III HD-Zip, KANADI, ARF3/4 (AUXIN RESPONSE FACTOR), YABBY and small non-coding RNAs [1–6]. Among these different types of regulators, YABBY family is specific to seed plants [7], and contains zinc finger-like and YABBY domains [8, 9]. The analysis of the zinc finger domain showed that it could work in protein-protein interactions for the formations of homo- and heterodimers as well as protein self-association [10]. Evolutionary analysis indicated that *YABBY* gene family consists of five members, including *FILAMENTOUS FLOWER/YABBY3* (*FIL/YAB3*), *YABBY2* (*YAB2*), *YABBY5* (*YAB5*), *CRABS CLAW* (*CRC*), and *INNER NO OUTER* (*INO*). In *Arabidopsis*, *FIL/YAB3*, *YAB2* and *YAB5* are expressed in the abaxial domain of lateral organs including cotyledons, leaves and floral organs, thus they were served as “vegetative *YABBY* genes”; whereas the other two (*CRC* and *INO*) are restrictedly expressed in the abaxial domains of carpels and the outer integument of ovules, respectively [11, 12].

Based on the discoveries over these years, many *YABBY* genes from different plant species have been shown to be involved in plant growth and development, particularly in lamina growth, establishment of leaf adaxial-abaxial polarity, SAM development and floral organ identity [11–16]. In *Arabidopsis*, *fil yab3* double mutant exhibited obvious phenotypes in the vegetative organs including linear cotyledons and leaves, abnormal vasculature and abaxial leaf surface, and ectopic SAM structures [12]; triple (*fil yab3 yab5*) and quadruple (*fil yab2 yab3 yab5*) mutants showed more severe phenotypes than the double mutant: diminutive and bushy plants lacking apical dominance and displaying a dramatic loss of lamina expansion and polarity defects in lateral organs [7]; while in *fil* single mutant, the flowers and floral organs were strongly affected, for example, increased sepals and carpels, missing petals, and radially symmetric stamens [11, 12, 17, 18]. Three *FIL*/*TOB* clade *YABBY* genes, *TONGARI-BOUSHI* (*TOB1*, *TOB2* and *TOB3*), were indicated to regulate spikelet and branch meristems in rice [19–21]. Rice *OsYABBY4* gene belonging to *FIL*/*YAB3* subfamily, exhibits possible functions in vasculature development [22], and regulates plant height, internode and floral organs development through modulating the gibberellin pathway [23]. *CtYABBY1*, a *FIL* homology, is sensitive to temperature variation and plays an important role in male sterility and fertility restoration in Chinese cabbage [15]. A YABBY-like gene *fasciated* (*fas*) from tomato (*Solanum lycopersicum*) regulates carpel number, fruit development and fruit size [24, 25]. Overexpressing an *Incarvillea arguta YAB2* subfamily gene *IaYABBY2* in *Arabidopsis* altered the adaxial-abaxial polarity of leaves and sepals, affected the development of florescence, and increased the anthocyanin content level and photosynthesis capability of plants after differential environment stress [26]. Two wild Chinese *Vitis pseudoreticulata* genes, *VpYABBY1* and *VpYABBY2*, belonging to *FIL* and *YAB2* subfamily, were shown to have divergent functions in the control of lateral organ development: *VpYABBY1* regulates leaf adaxial-abaxial polarity, while *VpYABBY2* may play an important role in carpel growth and grape berry morphogenesis [27]. Z*mYAB2.1*/*ZmSh1–1*, belonging to *YAB5* subfamily, was identified as a candidate gene controlling nonshattering ears in maize [28], and was also reported to interact epistatically with *teosinte-branched1* (*tb1*) to regulate the length of internodes within the ear [16]. In spearmint (*Mentha spicata*), a novel gene *MsYABBY5* (belonging to *YAB5* subfamily), was proved to be a repressor of secondary metabolism (terpene level) [29]. *Arabidopsis CRC* was reported to participate in the nectary development and carpel identity [8, 30]. In maize, drooping leaf (*drl*) gene, the homology of *Arabidopsis CRC*, was shown to regulate plant architecture through affecting leaf length and width, leaf angle, and internode length and diameter [31]. In rice, the drooping leaf gene (DL) not only regulates the leaf midrib formation, but also controls the specification of carpel in the flower [32–34]. The *Arabidopsis INO* was demonstrated to be necessary for polarity determination in the ovule [35].

Although many plant *YABBY* genes have been functionally studied, their roles in soybean are rarely reported. Zhao et al. [36] found soybean gene *GmYABBY10* might be a negative regulator of plant tolerance to drought and salt stress. In this study, a YABBY gene, designated as *GmFILa*, was isolated and functionally studied in soybean. Moreover, microarray analysis was performed to uncover the regulation mechanism of *GmFILa* in the transgenic *Arabidopsis*. Our results suggest the roles of soybean *GmFILa* in regulating leaf polarity development and potential functions in stress tolerance.

## Results

### Duplication pattern, phylogeny, gene structure, and expression analyses of soybean YABBYs

Until now, a total of 17 *YABBY* genes were identified in soybean [36]. Compared with *Arabidopsis* (5 members) [12], rice (*Oryza stative L*.) (8 members) [37], maize (*Zea mays L*.) (13 members) [38] and tomato (9 members) [39], soybean contains the most numerous members in *YABBY* gene family, which may be due to the two large-scale genome replications in soybean [40]. Therefore, we analyzed the duplication patterns of soybean *YABBY* genes and found that all *GmYABBY* genes were derived from segmental duplications without tandem duplications (Additional file 1: Figure S1 and Table S1). This suggests that segmental duplication might be the main cause of expansion in soybean *YABBY* family.

**Fig. 1:**
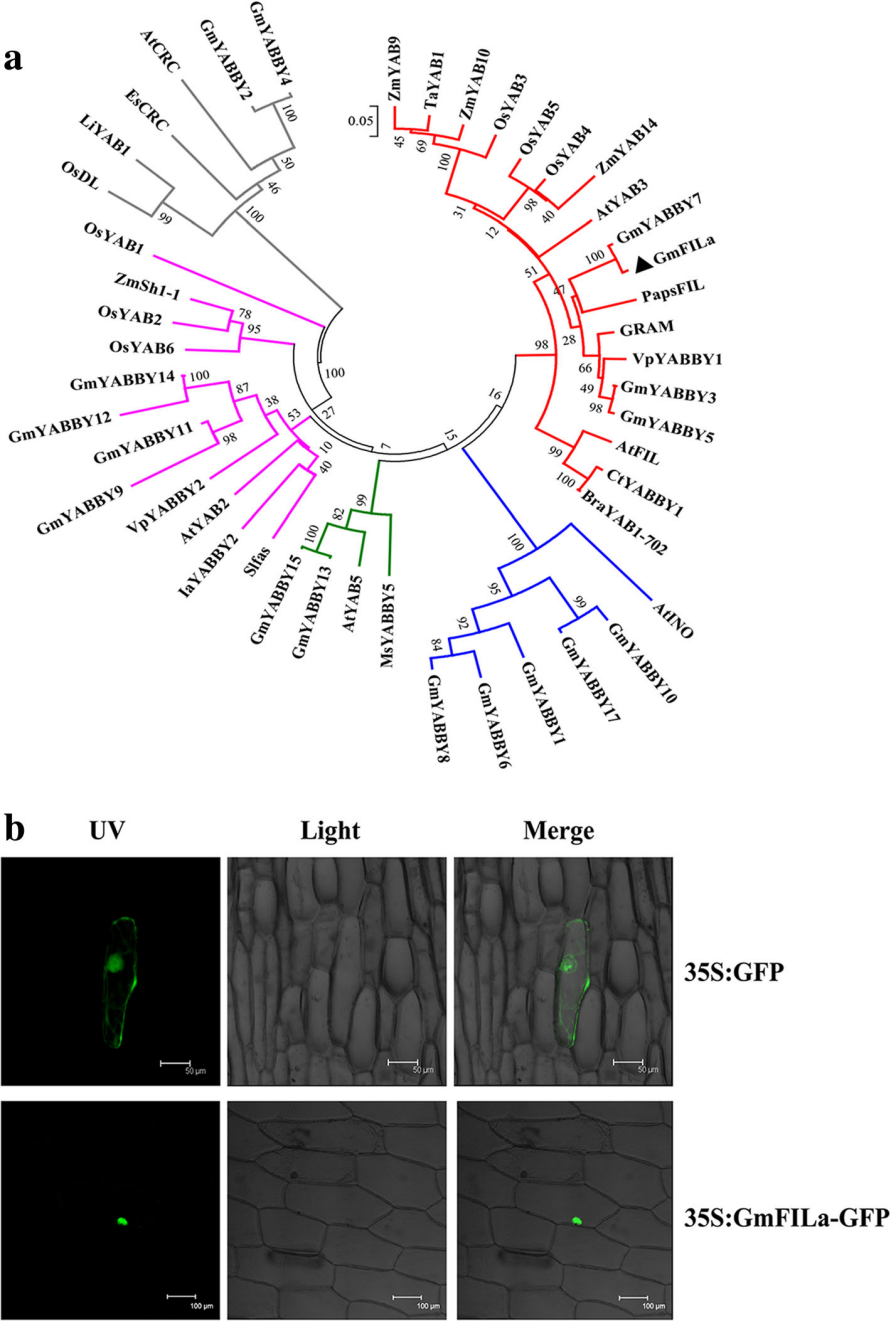
Phylogeny and subcellular localization of GmFILa. **a** Phylogenetic relationships of GmYABBYs with 29 YABBYs from other plants. Soybean YABBYs were named based on the previous report [36]. The phylogenetic tree was constructed with neighbor-joining method in MEGA 6.0 software and was divided into five subgroups marked with different colors. **b** GmFILa-GFP fusion protein and GFP alone were transiently expressed in onion epidermal cells, respectively. UV, images of GFP fluorescence; Light, bright field images of cell morphology; Merge, merged images. Scale bar was indicated in each panel

A neighbor-joining (NJ) phylogenetic tree was constructed based on soybean and 29 known YABBY proteins from monocotyledonous and dicotyledonous plants (Fig. 1a, Additional file 2: Table S4). As with the previous report that YABBY family consists of five subclasses including *FIL*/*YAB3*, *YAB2*, *YAB5*, *INO* and *CRC* [12], soybean *YABBY* gene family was also divided into five subgroups. As shown in the tree, *YABBY* genes from monocots and dicots clustered independently in each subgroup (Fig. 1a), indicating that they have functional differentiation. In addition, the functions of soybean *YABBY* genes could be inferred from the known plant *YABBY*s through phylogenetic relationships.

Exon-intron structure divergences usually represent the evolutionary relationships within gene families. In soybean *YABBY* gene family, exon number ranges from six to seven, and intron number is either five or six (Additional file 1: Figure S2). From the phylogenetic tree, most members within the same subgroup contain conserved exon/intron structures and similar gene lengths (Additional file 1: Figure S2), while genes in different subgroups show some differences.

Tissue expression profiles of 16 soybean *YABBY*s were gained based on the RNA sequencing (RNA-seq) data from SoyBase. Obviously, they were divided into two categories: the expression of *CRC* and *INO* is restricted to young leaf and/or flower; whereas “vegetative *YABBY* genes” have higher expression levels than *CRC* and *INO*, and express in most of the tissues, including leaf, flower, pod and seed, but not in root and nodule (Additional file 1: Figure S3). Expression profiles of eight *GmYABBY*s were analyzed in Plant Expression Database (Additional file 1: Figure S4). *FIL*/*YAB3* (containing *GmYABBY16/GmFILa*, *GmYABBY3* and *GmYABBY5*) and *YAB5* (*GmYABBY13*) members were shown to have abundant expression levels in SAM and axillary meristem, but low in non-apical meristem; by the contrast, *YAB2* (*GmYABBY9* and *GmYABBY12*) and *INO* (*GmYABBY1* and *GmYABBY8*) genes were found to be highly expressed in non-apical meristem (Additional file 1: Figure S4b). All eight genes have higher expression in sporophytic tissue compared with mature pollen (Additional file 1: Figure S4c). The expression in embryonic development showed that several *GmYABBY*s are highly expressed in young trifoliate leaf compared with other tissues during the globular and heart stages, and exhibit high expression in embryo proper at the cotyledon stage (Additional file 1: Figure S4d).

### Identification of *GmFILa*

A soybean curled-cotyledon mutant (*cco*), induced by sodium azide (NaN_3_) and ^60^Coγ ray from soybean cultivar Nannong 94–16, was previously identified in our group. Shi et al. [41] revealed that the transcript level of *GmYABBY16* is significantly increased in *cco* mutant compared to its wild type through the RNA-seq data analysis and semi-quantitative RT-PCR (sqPCR) examination. Thus, *GmYABBY16* was selected for functional characterization, especially on the regulation of cotyledon and leaf development. As *GmYABBY16* belongs to *FIL* subfamily, it was further named as *GmFILa*.

**Fig. 2:**
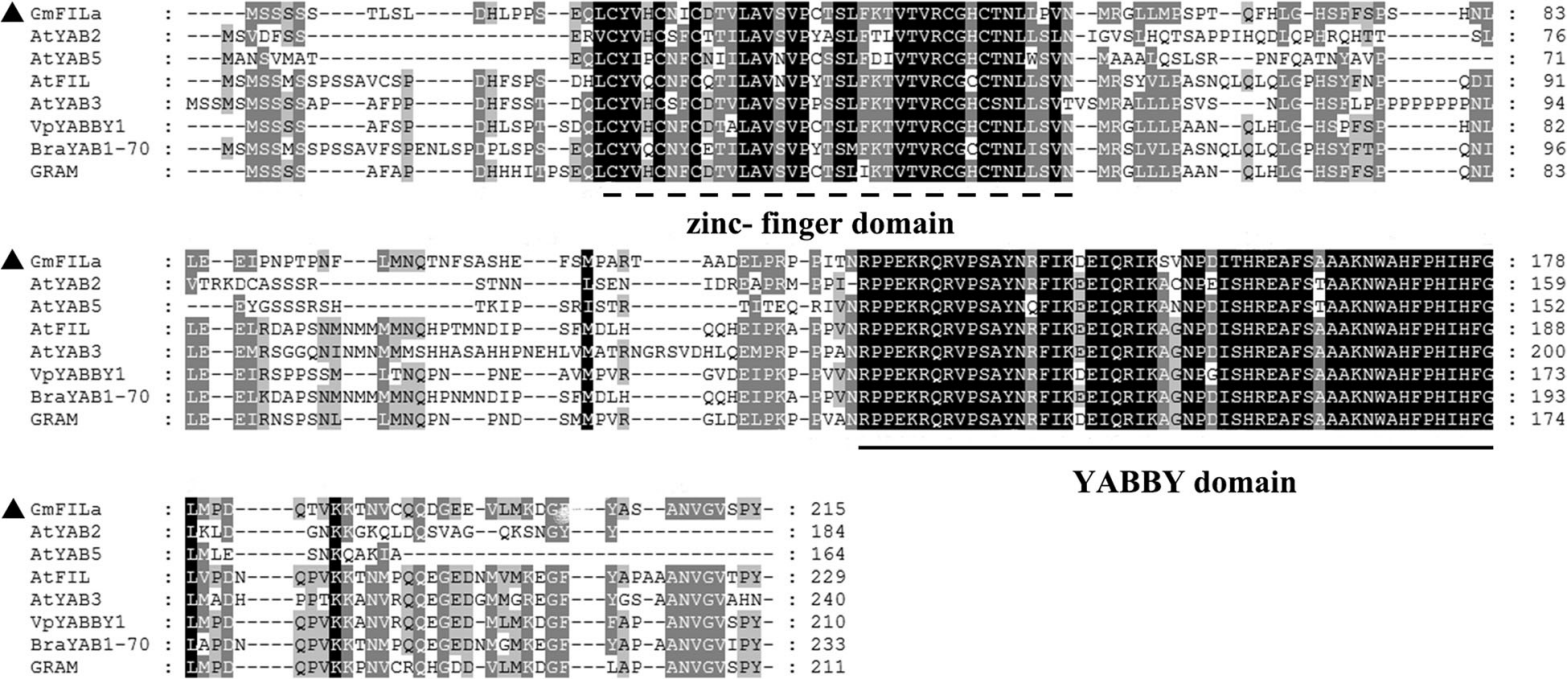
Alignment of plant YABBY protein sequences. GeneDoc software was used to show the Clustal alignment figure of soybean GmFILa and other YABBY orthologs from different plants. Conserved zinc-finger and YABBY domains are underlined with different lines. YABBY protein sequences are listed in Additional file 2: Table S4

The coding sequence (CDS) of *GmFILa* (*Glyma.17G138200*) was cloned via reverse transcription PCR (RT-PCR) from the leaf of soybean Nannong 94–16 cultivar (Additional file 1: Figure S5), which is 648 bp in length and encodes 215 amino acids along with a protein mass of 24.02 kDa and an isoelectric point (PI) of 7.16. Physicochemical properties analysis revealed that GmFILa is a hydrophilic (with GRAVY value of − 0.341) and unstable protein (with instability index of 52.46). The GmFILa protein was predicted to have conserved YABBY domain in the N-termini and a zinc finger-like motif in the C-termini by alignments with several other plant YABBY proteins (Fig. 2). Phylogenetic analysis showed that GmFILa was grouped together with PapsFIL (identity of 67.53%) from *Papaver somniferum*, VpYABBY1 (identity of 72.56%) from wild Chinese *Vitis pseudoreticulata* and GRAM (identity of 75%) from *Antirrhinum majus* (Fig. 1a). Among these orthologs, *PapsFIL* was shown to regulate highly lobed leaf patterning [42]; overexpression of *VpYABBY1* in *Arabidopsis* caused the partial abaxialization of the adaxial epidermises of leaves [27]; and *GRAM* promotes lateral growth and abaxial cell fate in the growing leaf primordia [13].

### GmFILa is a nuclear-localized protein

To obtain 35S:GmFILa-GFP, the coding region of *GmFILa* was fused to the green fluorescent protein (GFP) reporter gene which is under the control of the cauliflower mosaic virus (CaMV) 35S promoter. Further, the recombinant construct and empty vector (35S:GFP) were transformed into onion epidermal cells, respectively. Confocal images revealed that the GmFILa-GFP fusion protein was localized exclusively to the nucleus, by contrast, the empty vector was uniformly distributed throughout the whole cell (Fig. 1b). This observation indicated that GmFILa is a nuclear-localized protein, implying that GmFILa, like other YABBYs [22, 27], functions as a transcription factor.

### Tissue expression pattern analysis of *GmFILa*

**Fig. 3:**
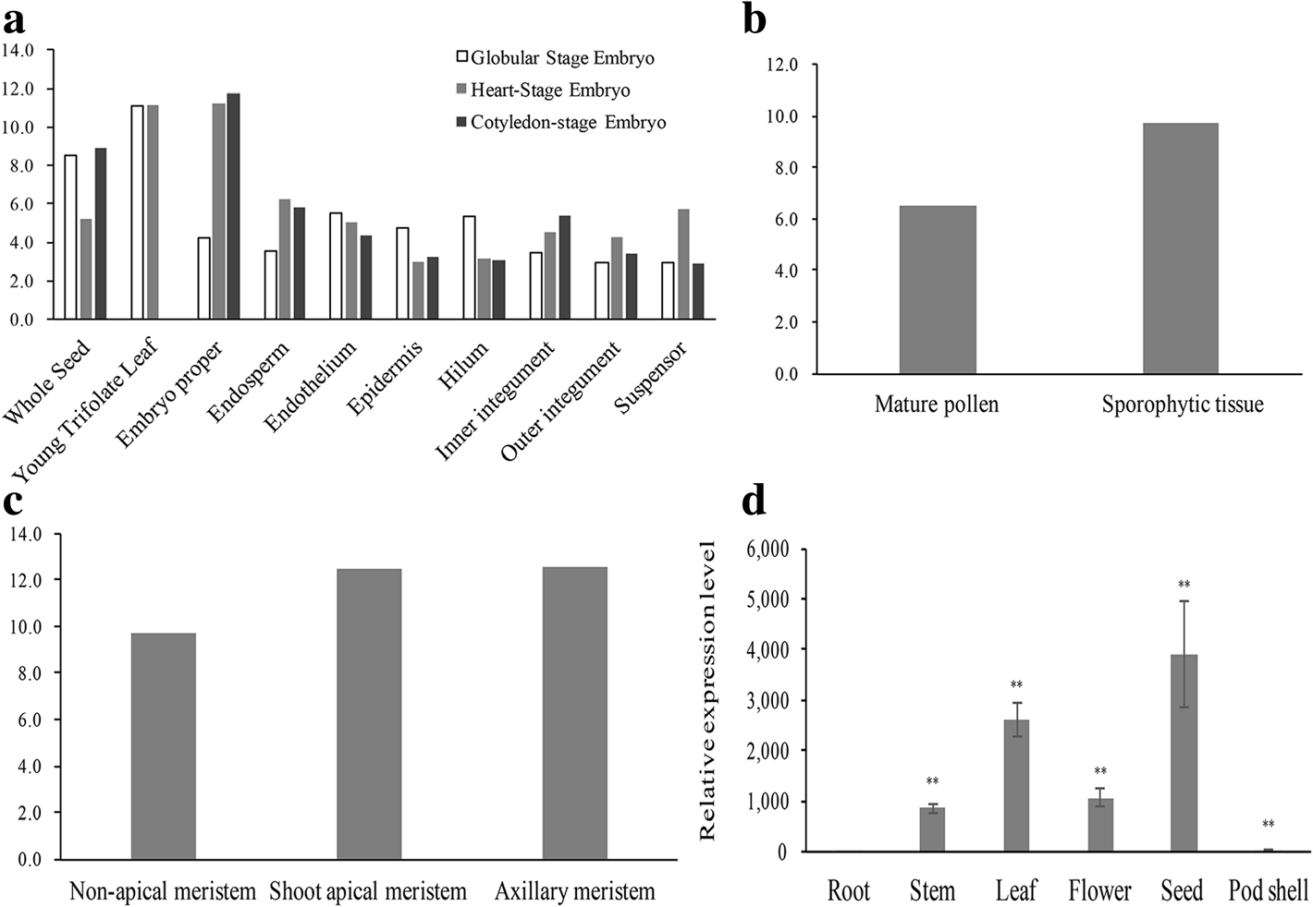
Tissue-specific expression analysis of *GmFILa*. **a**-**c** Digital expression of *GmFILa* in different tissues from microarray data [70]. The Y-axis represents the log2 ratios for the MAS-normalized values. **d** Tissue-specific expression profiles of *GmFILa* with qRT-PCR amplification in seedling root, stem, leaf, flower, seed and pod shell. The expression of *GmFILa* in root was used as a control (expression value = 1). Paired-samples *t*-test (two-tail) was selected for statistical analysis. * 0.01 < *P* < 0.05; ** *P* < 0.01

From RNA-seq data, *GmFILa* is highly expressed in leaf, followed by seed, flower, and pod tissues, but not in root and nodule (Additional file 1: Figure S3). The qRT-PCR examination indicated that *GmFILa* has the highest expression level in seed at 20 days after flowering (DAF), followed by leaf, flower, stem, pod shell (20DAF) and root (Fig. 3d). This discrepancy might be due to the differences in the RNA samples (*G. max* A81–356022 for RNA-Seq and Nannong 94–16 for qRT-PCR) or methods. Based on microarray data, we analyzed the detailed expression of *GmFILa* in the tissues of seed, flower, and meristem (Fig. 3a-c). During seed embryo development, *GmFILa* has relatively high expression in whole seed, young trifoliate leaf and embryo proper compared with other tissues. At globular stage, *GmFILa* has the highest expression in young trifoliate leaf; at heart stage, the expression decreases in whole seed and increases in embryo proper; at cotyledon stage, the expression of *GmFILa* is increased in whole seed, but absent in trifoliate leaf (Fig. 3a).

**Fig. 4:**
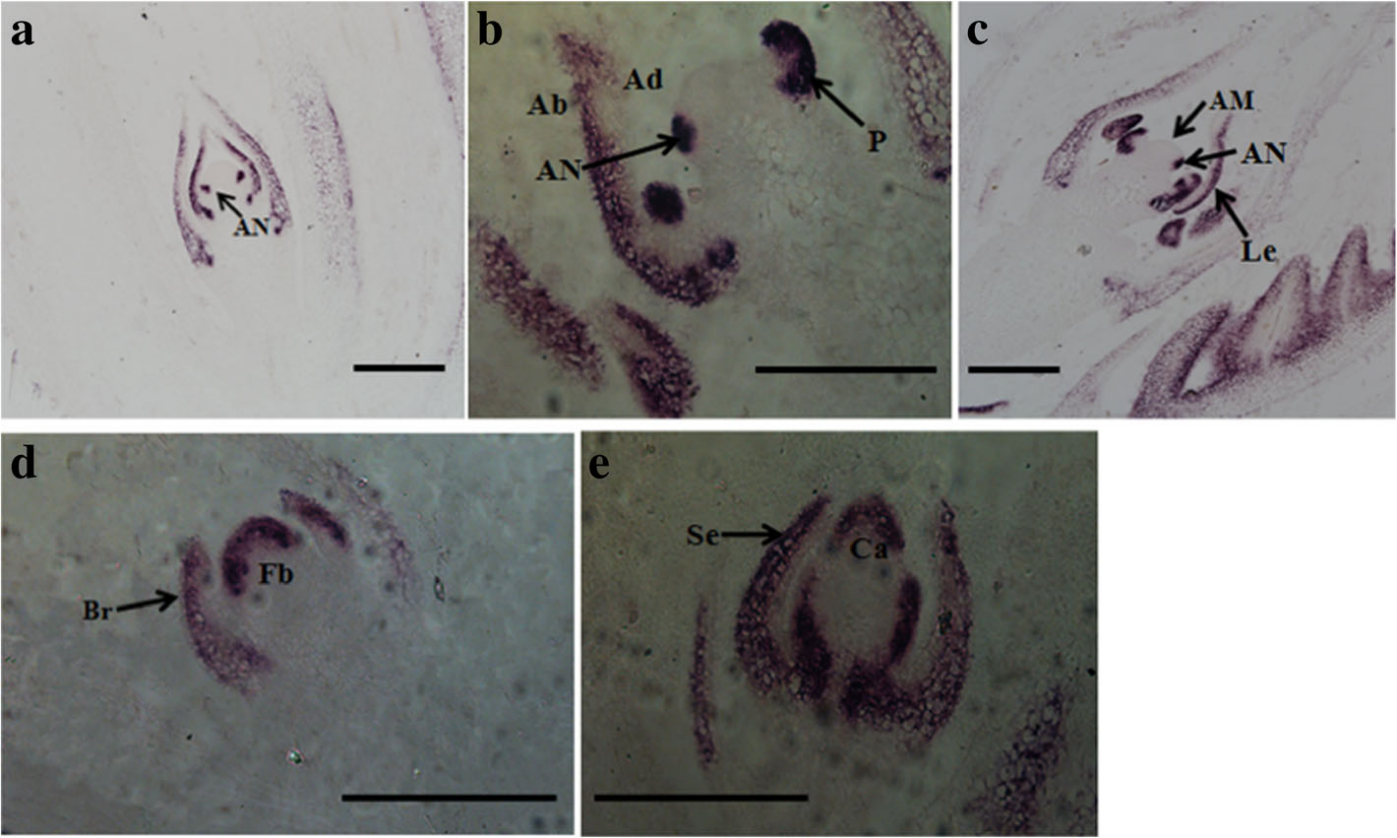
In situ hybridization of *GmFILa* in soybean leaves and flowers. **a**-**c** The longitudinal sections of leaf primordia. **d**-**e** The longitudinal sections of flower bud primordia. AN, leaf anlagen; Ab, abaxial; Ad, adaxial; P, leaf primordia; AM, apical meristem; Le, developing leaf; Br, bract; Fb, flower bud primordia; Se, sepal; Ca, carpel primordia. Bars:100 μm

The mRNA in situ hybridization was employed to precisely examine the expression of *GmFILa* in soybean leaf and flower tissues (Fig. 4). Before the complete formation of the leaf primordia, two incipient leaf primordia are formed on both sides of the apical meristem (AM). First, the transcripts of *GmFILa* were distributed throughout the incipient leaf primordia (Fig. 4a); further, *GmFILa* was gradually expressed in abaxial cells with the development of leaf primordia (Fig. 4a-c). During flower bud differentiation, *GmFILa* was mainly expressed at the top of the flower bud primordia, carpel primordia, abaxial domains of bract and sepal (Fig. 4d, e). These expression patterns suggested that *GmFILa* plays an important role in the stimulation of lateral organs and subsequent growth of abaxial region.

### Promoter cis-elements prediction and inducible expression analysis of *GmFILa*

The sequence analysis in *GmFILa* promoter region showed some *cis*-acting elements related to drought, light, and hormone (such as auxin and gibberellic acid) responses (Table 1). Besides, Dof transcription factor binding site was also found in the promoter region. Collectively, *GmFILa* might be induced by a variety of regulatory factors associated with stresses or hormones.

**Table 1:** Sequence analysis of *GmFILa* promoter

Name	Number	Sequence	Function
ABRELATERD1	1	ACGTG	Drought response
ACGTATERD1	2	ACGT	Drought response
ARFAT	1	TGTCTC	Auxin response
DOFCOREZM	32	AAAG	Dof binding site
GT-motif	2	GTGTGTGAA/GGTTAA	Light response
GARE-motif	1	AAACAGA	Gibberellin response
TCA-element	1	CAGAAAAGGA	Salicylic acid response

**Fig. 5:**
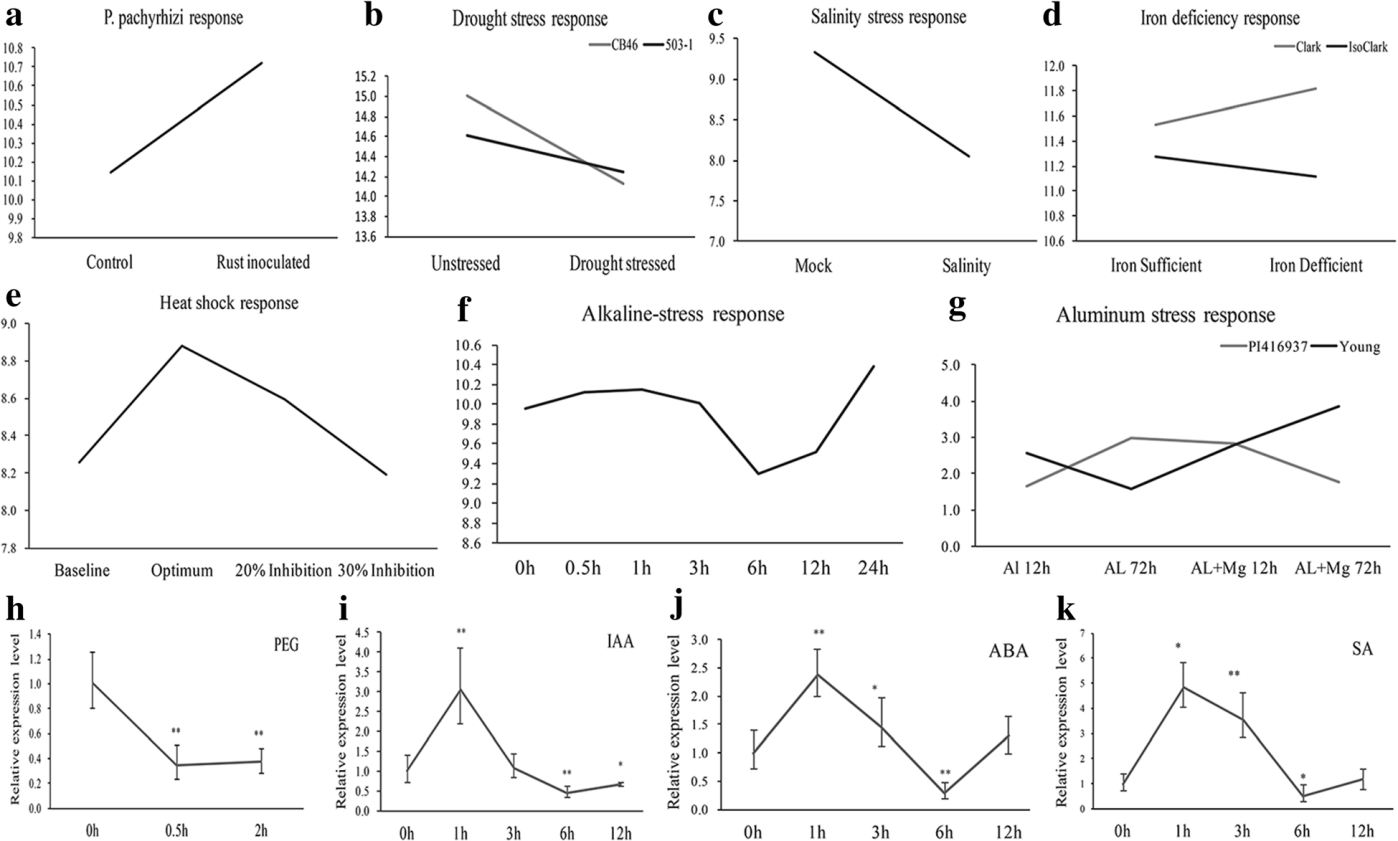
Induction expression analysis of *GmFILa*. **a**-**g** Digital expression of *GmFILa* in different treatments from microarray data [70]. Y-axis represents the log2 ratios for the expression values. **h**-**k** qRT-PCR examination of *GmFILa* in response to drought (PEG) and three hormone treatments (IAA, ABA and SA). The expression of *GmFILa* at 0 h was used as a control (expression value = 1). Paired-samples *t*-test (two-tail) was selected for statistical analysis. * 0.01 < *P* < 0.05; ** *P* < 0.01

By investigating the microarray data, we found that the expression of *GmFILa* was up-regulated in leaf tissue after *P.pachyrhizi* inoculation (Fig. 5a) for 72 h. And its expression was down-regulated when treated with abiotic stresses like drought, salt and heat shock (Fig. 5b, c, e). Other stresses including metal ions (Fig. 5d, g) and alkaline (Fig. 5f) treatments could also induce the expression of *GmFILa*. Further, qRT-PCR was used to investigate the expression of *GmFILa* exposed to several treatments. As shown in Fig. 5h-k, *GmFILa* could be induced by polyethylene glycol (PEG), indole acetic acid (IAA), abscisic acid (ABA) and salicylic acid (SA), implying that *GmFILa* might be involved in responses to both stress and hormone treatments.

### Phenotype investigation of *Arabidopsis* transformed with soybean *GmFILa*

**Fig. 6:**
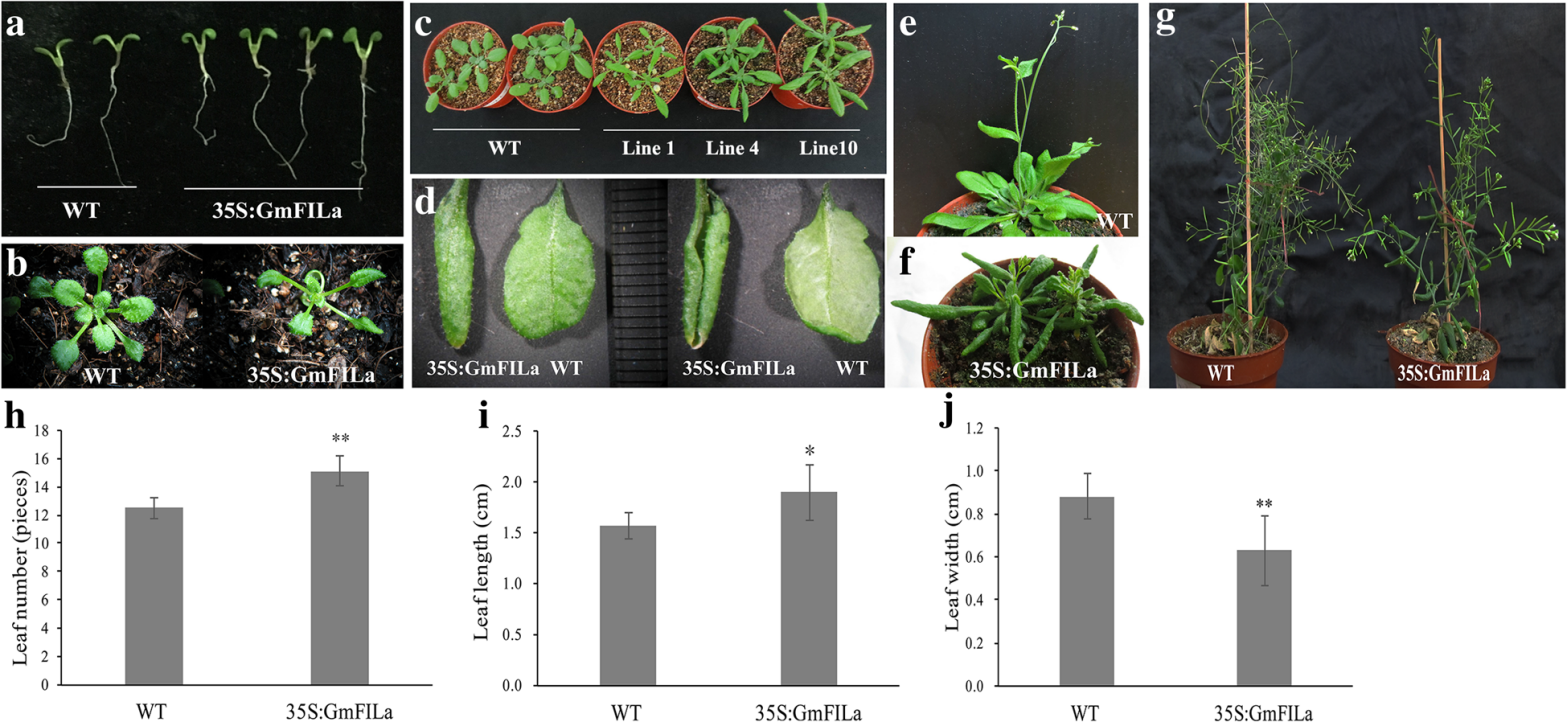
Phenotypic analysis of *GmFILa* transgenic *Arabidopsis* plants. **a** The cotyledons of 10-day-old seedlings. **b** The leaves of 15-day-old seedlings. **c** The leaves of 25-day-old seedlings. **d** The phenotype of adaxial (left) and abaxial (right) axis of leaves. **e**-**f** Phenotype of 41-day-old wild and *GmFILa* transgenic plants. **g** Phenotype of *GmFILa* transgenic and WT plants during fruiting stage. **h** Leaf number statistics of the rosette leaves of 35-day-old seedlings. **i** Leaf length statistics of the rosette leaves of 35-day-old seedlings. **j** Leaf width statistics of the rosette leaves of 35-day-old seedlings. WT: Wild type; 35S:GmFILa: transgenic *Arabidopsis* with *GmFILa* gene. Paired-samples *t*-test (two-tail) was selected for statistical analysis. * 0.01 < *P* < 0.05; ** *P* < 0.01

The CDS of *GmFILa* was cloned into pBI121 vector to generate the construct 35S:GmFILa, and then the recombinant construct was transferred into the *Arabidopsis* using *Agrobacterium*-mediated transformation method. A total of 10 transgenic lines were obtained and three of which with relatively higher expression levels (Additional file 1: Figure S6) were thus further used for phenotype investigation. Compared with wild-type (WT) plants, all homozygous transgenic lines (10-day-old) from T_4_ generation exhibited outward curled cotyledons (Fig. 6a); then the growing leaves were curled from the adaxial side to abaxial side and became long-narrow (Fig. 6b-d), this phenotype became more obvious with the increase of the leaf ages (Fig. 6b-d). Further, measurement of leaf traits with 35-day-old seedlings indicated that the leaf number and length of transgenic *Arabidopsis* plants were significantly increased, while the leaf width was significantly reduced (Fig. 6h-j) compared with WT. In addition, the SAM of transgenic plants was slightly inhibited (Fig. 6e, f), and the flowering stage was clearly delayed (Fig. 6e, f); however, the plants can eventually bear fruits (Fig. 6g).

**Fig. 7:**
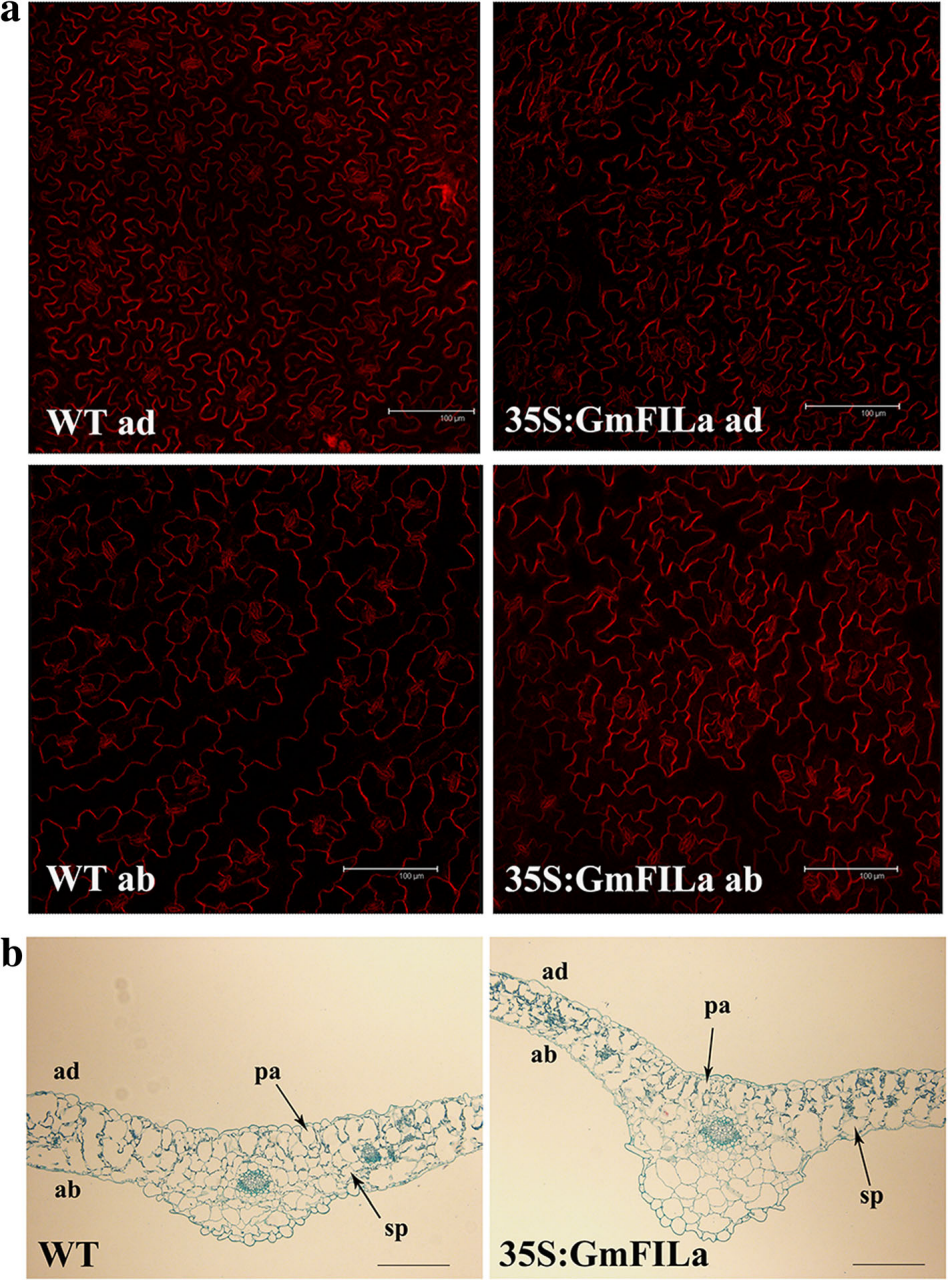
Histological analysis of rosette leaves of WT and 35S:GmFILa transgenic *Arabidopsis*. **a** Observation of leaf epidermal cells in WT and *GmFILa* transgenic *Arabidopsis* plants. ab, abaxial epidermal cells; ad, adaxial epidermal cells. Bars: 100 μm. **b** Observation of the transverse section of rosette leaves in WT and 35S:GmFILa plants. ad, adaxial; ab, abaxial; pa, palisade mesophyll; sp., spongy mesophyll. Bars: 50 μm

As the morphological development of multicellular organisms depends on the cell morphology in the tissue layer, such as pavement cells of leaf epidermises, we thus initiated to compare the epidermal cell morphology of transgenic and WT plants. In the WT plants, the adaxial epidermal cells of rosette leaves were regular and uniform, while the abaxial cells were quite irregular (Fig. 7a). However, unlike WT, the adaxial surfaces of 35S:GmFILa transgenic plants are similar to the abaxial surfaces with irregular cell shapes, greatly varied cell sizes and disordered arrangement (Fig. 7a). Therefore, it may be concluded that the changes in epidermises led to the leaf curling phenotype in *GmFILa* transgenic *Arabidopsis*. Further, the paraffin section examination showed that 35S:GmFILa plants contained normal cell layers in mesophylls as with WT, indicating that overexpression of *GmFILa* in *Arabidopsis* does not affect the internal structure of leaves (Fig. 7b). Conclusively, we demonstrate that overexpression of soybean *GmFILa* causes partial abaxialization of adaxial leaf epidermises in *Arabidopsis*.

### Microarray analysis of *GmFILa* transgenic plants

**Fig. 8:**
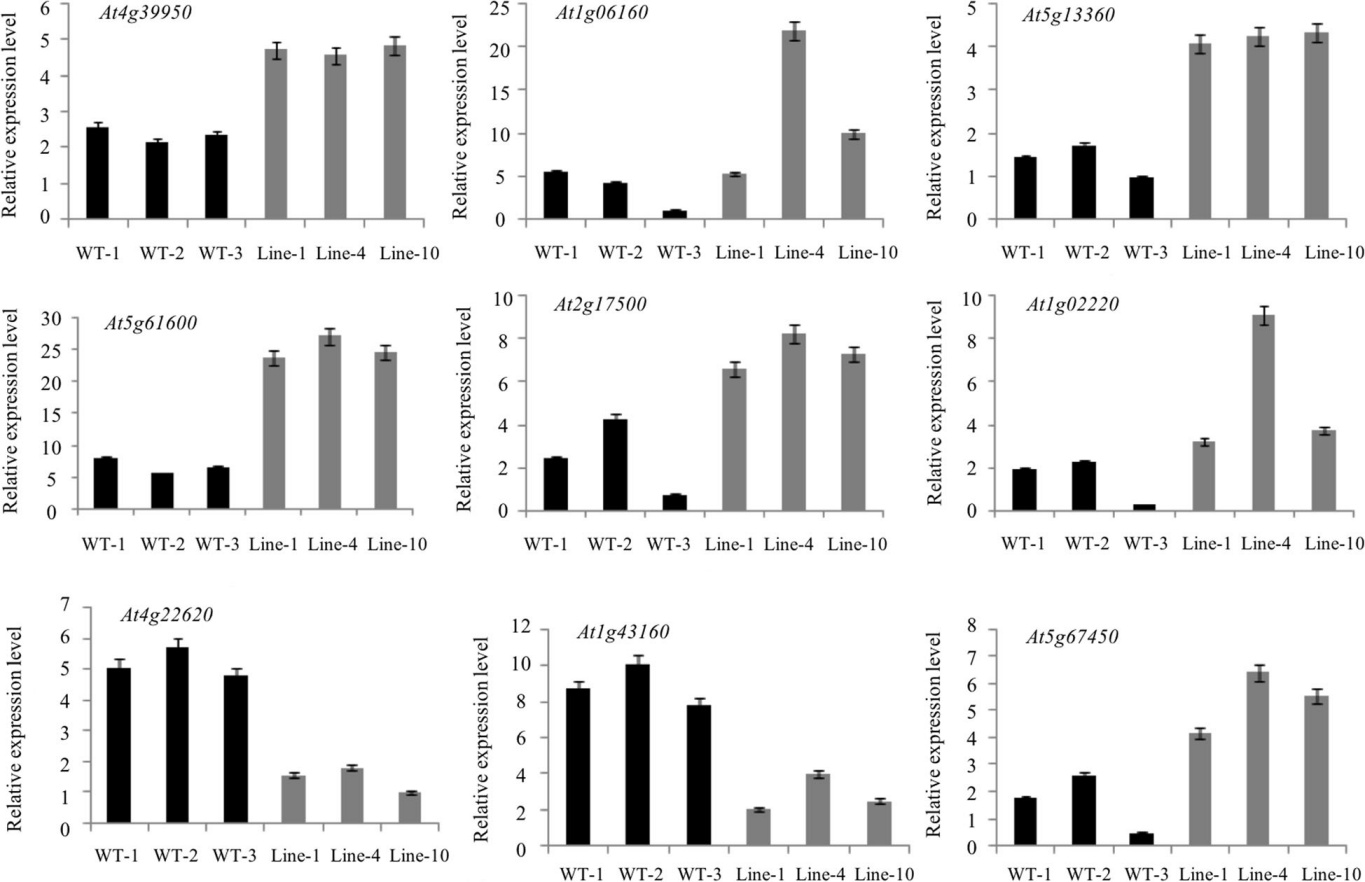
Verification of microarray data results using qRT-PCR. Nine DEGs were selected for confirming their relative expression levels in leaf tissue of GmFILa transgenic and WT *Arabidopsis* plants. Paired-samples *t*-test (two-tail) was used. * 0.01 < *P* < 0.05; ** *P* < 0.01

Leaf transcriptomes of WT and *GmFILa*-overexpressing *Arabidopsis* plants were compared through microarray analysis. To validate the reliability of microarray data, nine probe sets (genes) were selected for qRT-PCR examination (Fig. 8). Our results showed that *At4g39950*/*CYP79B2* (auxin biosynthesis), *At5g13360* (auxin-responsive protein), *At5g61600*/*ERF104* (ethylene response factor), *At2g17500*/*PILS5* (auxin efflux carrier family protein), *At1g02220*/*ANAC003* (NAC domain containing protein) and *At5g67450*/*AZF1* (zinc-finger protein), were all up-regulated in 35S:GmFILa plants compared with WT, whereas *At4g22620* (SAUR-like auxin-responsive protein) and *At1g43160*/*RAP2.6* (ethylene response factor) were down-regulated, suggesting that these hormone biosynthesis and signal transduction related genes were directly or indirectly regulated by GmFILa. The relative expression levels of most examined genes generally agreed with the microarray data except for *At1g06160*, which had different expression changes in transgenic line 1 (Fig. 8).

**Fig. 9:**
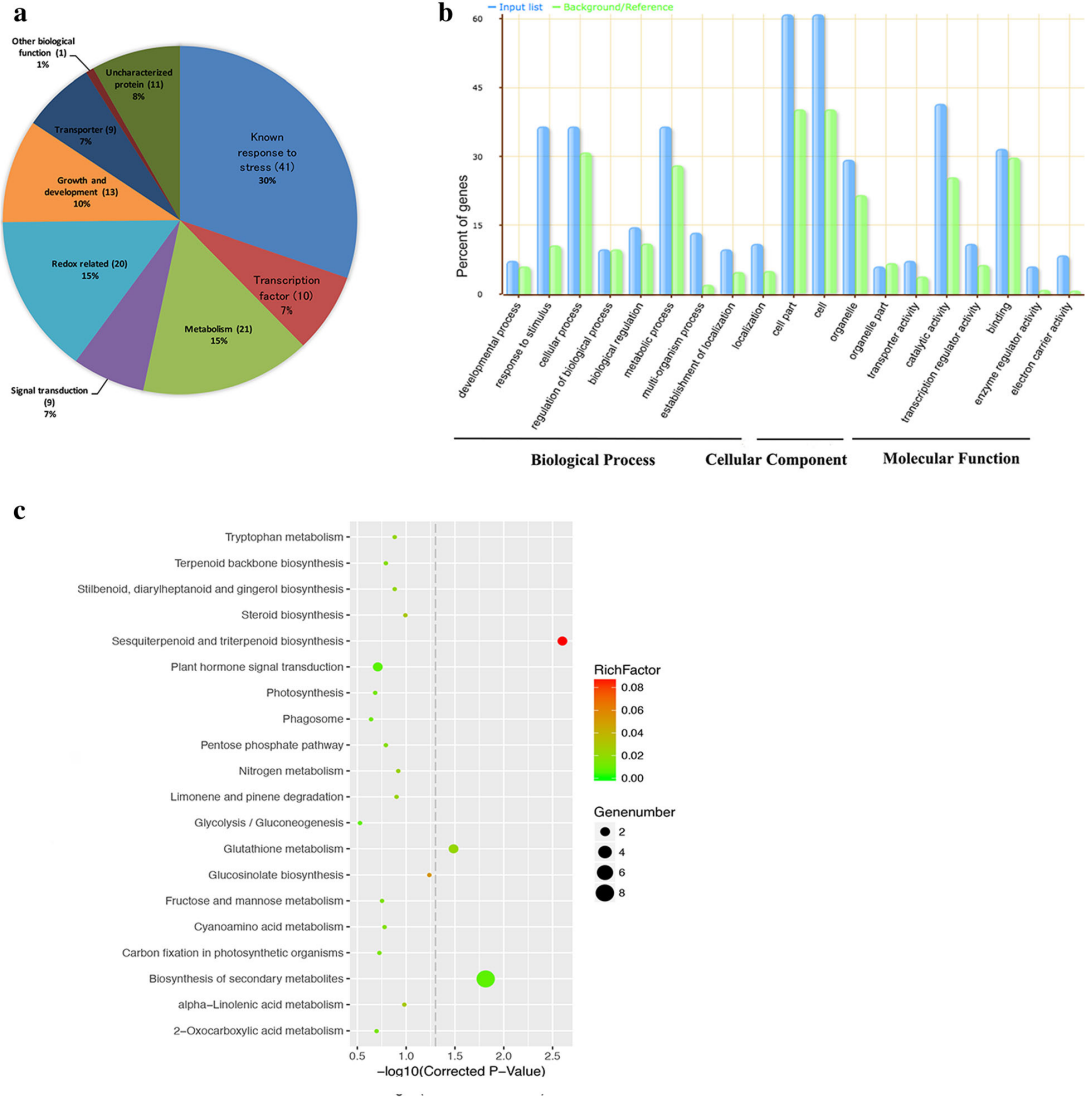
Cluster analysis of DEGs in wild type and transgenic *Arabidopsis* plants based on microarray data. **a** The pie chart represents differentially regulated (*P*-value < 0.05 and |FC| ≥ 2) genes in different functional categories. Gene number was indicated in parenthesis. When some genes obtain more than one functional category, they would be counted in every corresponding category. Details were listed in Additional file 3: Table S5. **b** Gene Ontology classification of the DEGs between transgenic and WT plants. The X-axis is the definition of GO terms, and Y-axis is the percentage of genes mapped by the GO term. The blue represents “input list” and refers to DEGs; the green represents “background or reference,” which means background genes. The percentage for the input list is calculated by the number of DEGs mapped to the GO term divided by the number of all DEGs in the input list. The same calculation was applied to the reference list to generate its percentage. Details provided in Additional file 4: Table S6. **c** The KEGG enrichment pathways of DEGs in *GmFILa* transgenic *Arabidopsis* plants. The X-axis represents the enrichment degree, which is determined by the correct *P*-values; the Y-axis represents the pathway terms; the color of the spots indicates the enrichment factor, which represents the ratio of number of DEGs compared with genome background in a pathway, and the spots size represents the number of significant DEGs. The picture was drawn by local program. Details provided in Additional file 5: Table S7

With *P*-value < 0.05 and |FC| ≥ 2, a total of 82 probe sets exhibited significant changes in transgenic lines, with 62 being up-regulated and 20 down-regulated, suggesting that transforming *GmFILa* into *Arabidopsis* affected expression of a number of endogenous genes (Additional file 3: Table S5). Based on the gene annotation, these DEGs could be grouped into different functional categories: transcription factors, transporters, and genes involved in growth and development, metabolism, signal transduction, redox reaction and stress response (Fig. 9a). Functional analysis showed that 13 (10%) of the DEGs were related to growth and development, including eight up-regulated genes and five down-regulated genes. *At4g30410*/*IBL1* and *At1g58340*/*AtZF14*, which belongs to bHLH transcription factor and MATE transporter gene family, respectively, were all reported to negatively regulate plant cell elongation in *Arabidopsis* [43, 44]. *At2g06850*/*AtXTH4* was demonstrated to have possible role in cell wall rigidification [45]. *At2g37430*/*ZAT11*, *At2g22850*/*bZIP6* and *At1g53700/WAG1* are several genes related to plant root development [46–48]. A NAC transcription factor gene, *At3g15510*/*NAC2*, not only regulates lateral root, flower and embryogenesis developments, but also responds to salt stress [49–52]. Several DEGs involved in auxin (*At4g39950*/*CYP79B2*; *At2g17500*/*PILS5*) [53, 54], jasmonic acid (*At3g55970*/*ATJRG21*) [55] and ethylene (*At1g06160*/*ORA59*; *At5g61600*/*ERF104*) [56, 57] signal pathways were up-regulated in *GmFILa* overexpressing *Arabidopsis*. Some metabolic pathway-related DEGs were up-regulated in transgenic *Arabidopsis* including *At2g29470*/*GSTU3* (glutathione transferase) [58], *At5g22300*/*NIT4* (nitrilase) [59] and *At5g27420*/*CNI* (ubiquitin ligase) [60]. Many genes related to biotic/abiotic stresses were also found to be up-regulated in transgenic *Arabidopsis*, such as *At4g11650*/*AtOSM34* [61], *At2g35980*/*AtNDR1* [62], *At3g15510*/*AtNAC2* [51], and *At5g67450*/*AtZF1* [63].

Further, gene ontology (GO) and kyoto encyclopedia of genes and genomes (KEGG) analyses were used to identify the key processes for transgenic plants. 82 DEGs had corresponding GO annotations (Fig. 9b) and were significantly enriched in 18 GO terms (corrected *P*-value < 0.05; FDR) (Additional file 4: Table S6). Of the 18 GO terms, most of the DEGs’ encoded products were associated with “cell part” and “cell”, followed by “catalytic activity” and “stress response”, indicating that many of the DEGs might be involved in plant growth and development. Furthermore, 41 DEGs were classified as associated with 25 relevant KEGG pathways (Additional file 5: Table S7), which are directly or indirectly essential for plant growth and development. A total of three functional KEGG pathways were significantly enriched (P-value < 0.05) (Fig. 9c) including biosynthesis of secondary metabolites, sesquiterpenoid and triterpenoid biosynthesis, and glutathione metabolism. There were eight genes involved in secondary metabolism pathway. The nitrilase (nitrile aminohydrolase) hydrolyze indole-3-acetonitrile to the phytohormone indole-3-acetic acid in vitro, and the sites of nitrilase expression may represent the sites of auxin biosynthesis in *Arabidopsis* [64]. In our microarray data, one nitrilase gene (*NIT4*) was up-regulated, suggesting that auxin synthesis might be also influenced in transgenic plants. *At4g39950*/*CYP79B2* (cytochrome P450), a critical enzyme in auxin biosynthesis in vivo, was up-regulated in *GmFILa* transgenic plants. Plants overexpressing *CYP79B2* contain increased free auxin levels and thus exhibit a series of auxin overproduction phenotypes including long hypocotyls and epinastic cotyledons [53].

## Discussion

### Tissue expression patterns of soybean *YABBY* genes

Many plant *YABBY* genes have been studied to reveal their functions in regulating plant leaf [42], meristem [19, 65], flower organ [30], and fruit [25] development. As the tissue-specific gene expression pattern, to some extent, can reflect their potential functions, we thus investigated the expression patterns of all soybean *YABBY* genes in various tissues. Digital expression data showed that there existed two types of tissue expression characteristics among soybean *YABBY* genes. *INO* and *CRC* members were only expressed in several specific tissues and exhibited relatively lower expression level compared with the vegetative *YABBY* genes. This result suggests that the differential expression characteristics of *YABBY* genes may determine their different functions as shown in the phylogenetic tree. Duplicated genes tend to share common or similar functions, thus the tissue-specific expression of segmental duplicated genes was compared. Three pairs of duplicated genes (*GmYABBY2* and *GmYABBY4*, *GmYABBY13* and *GmYABBY15*, and *GmYABBY9* and *GmYABBY11*) were all shown to have similar tissue expression patterns, which may indicate their functional redundancy.

### Soybean *GmFILa* is involved in the establishment of abaxial-adaxial polarity in *Arabidopsis* leaf

*YABBY* gene family is responsible for the development of abaxial cell fate in lateral organs of *Arabidopsis*. Many *YABBY* genes in other plant species have been suggested with their functions in different various processes of growth and development [12, 14, 23, 26]. However, much less is reported about the functions of soybean *YABBY* genes during developmental processes. *GmFILa*, belonging to *FIL*/*YAB3* subgroup, is closely clustered with three known *YABBY* genes (*PapsFIL*, *VpYABBY1* and *GRAM*), which are all associated with leaf polarity and morphology development [13, 27, 42], suggesting that *GmFILa* may possess similar function to these homologs in leaf growth regulation. In situ hybridization analysis suggested that *GmFILa* was expressed in abaxial cell layers with the development of leaf primordia. Overexpressing *GmFILa* in *Arabidopsis* produced narrow and curled leaf morphology via altering the adaxial-abaxial polarity. This phenotype was also observed in *Arabidopsis* plants transformed with *OsYABBY4* from rice [22], *VpYABBY1* from *Vitis pseudoreticulata* [27], and *BraYAB1–702* from Chinese cabbage [65]. These results may suggest that *FIL* homologs in angiosperm gained the conserved functions in the regulation of leaf development and the establishment of abaxial-adaxial polarity.

### *GmFILa* might be involved in the development of soybean *cco* mutant

Soybean *cco* mutant shows a series of aberrant phenotypes compared with its wild type, including curled cotyledons, longer growth periods, reduced root systems, and small plants [41, 66]. RNA-seq and sqPCR analysis suggested that the transcript level of *GmFILa* was significantly increased in *cco* mutant than wild type in pod tissues at 7 DAF [41]. Also, our experiments showed that 35S:GmFILa transgenic *Arabidopsis* altered the cotyledons and leafs morphology, and delayed the flowering. These results, indirectly, indicate that *GmFILa* might be involved in the soybean *cco* mutant development, particularly in the regulation of cotyledon development and growth period.

### Overexpression of *GmFILa* altered the expression of genes involved in growth and development

We used microarray data to explain the mechanism by which *GmFILa* affects the morphological phenotypes in transgenic *Arabidopsis* (Additional file 6: Table S8). The results showed that overexpression of *GmFILa* significantly altered the expression of a series of endogenous genes in *Arabidopsis*. There were 82 genes in total found to be significantly differentially expressed in transgenic lines. These differentially expressed genes were involved in growth and development, metabolism, signal transduction, redox reaction and stress response. Some of important growth and development related genes are worth mentioning, include transcription factors (ERF/AP2 transcription factor, NAC domain containing protein, C2H2 and C2HC zinc fingers superfamily protein) [46, 51], signal transduction components (protein-serine/threonine kinase, thioredoxin superfamily protein) [48, 67], hormone regulator (auxin synthesis protein, jasmonate-regulated protein) [53, 55], transporter (auxin efflux carrier family protein, MATE efflux family protein) [44, 54] and metabolism participants (nitrilase, RING type ubiquitin ligase) [59, 60]. *At5g16440*/*IPP1*, the isopentenyl/dimethylallyl diphosphate isomerase (IPI), was found to be up-regulated in transgenic *Arabidopsis*. IPI, catalyzing the interconversion of isopentenyl diphosphate (IPP) and dimethylallyl diphosphate (DMAPP), was reported to regulate plant growth by using *IPI*-defective mutants: loss of two *IPI* genes confer dwarfism and male sterility in *Arabidopsis* plants under long-day conditions [68]. Taken together, *GmFILa* likely affects transgenic *Arabidopsis* phenotypes via affecting the expression of genes in different biological processes.

Interestingly, microarray data indicated that many DEGs (30%) in *GmFILa* transgenic plants were involved in stress response, suggesting that *GmFILa* might regulate the stress tolerance. Moreover, promoter *cis*-acting elements analysis, digital expression data and qRT-PCR all revealed that *GmFILa* could be induced by drought stress, we thus conducted drought experiment using 25-day-old WT and *GmFILa* transgenic plants. However, after 2 weeks of drought treatment, no significant differences in phenotypes were detected between WT and transgenic plants, all of them began to turn yellow and purple (Additional file 1: Figure S7). Therefore, more experiments involving drought and other abiotic stresses need to be conducted for verification.

## Conclusion

In summary, our work provides the first insight into the functional role of soybean *GmFILa* gene. Ectopic expression of *GmFILa* causes alteration in leaf polarity, SAM development and flowering time; and influences the expression of 82 genes of different biological processes in *Arabidopsis* leaf. *GmFILa* might also be involved in plant stress tolerance. Further study with soybean transgenic plants will help us to gain a better understanding of the function of *GmFILa*.

## Methods

### Plant materials

Soybean seeds from cultivars Nannong 94–16 and Williams82 were provided by Soybean Research Institute, Nanjing Agricultural University, China. The ecotype Columbia-0 (Col-0) of *Arabidopsis thaliana*, kept in our laboratory, was used as wild type (WT).

### Plant growth conditions

The soybean seeds (Nannong 94–16) were grown under field conditions at Jiangpu experimental station, Nanjing Agricultural University, Nanjing, China. Different tissues from various developmental stages were used to examine the expression pattern of *GmFILa*. Roots, stems and leaves were collected at the third euphylis expanding stage. Mature flowers were sampled at flowering stage. Seeds and pod shells were harvested at 20 days after flowering (DAF). All the samples were frozen in liquid nitrogen and then stored at − 80 °C for later RNA extraction.

*Arabidopsis* seeds were first incubated for 48–72 h at 4 °C and then grown in a growth room under conditions of 16/8 h light/dark, 23/22 °C, with 70% relative humidity. The leaf number, leaf length and leaf width of transgenic and wild *Arabidopsis* plants were measured using 35-day-old seedlings; 15 plants of each genotype were analyzed.

### Isolation of the *GmFILa* gene

The coding sequence (CDS) of *GmFILa* was isolated from leaf tissue of soybean cultivar Nannong 94–16 via reverse transcription PCR (RT-PCR) with specific primers (Additional file 1: Table S2). Then the resulting fragment was cloned into pMD19-T vector (Vazyme, Nanjing, China) and sequenced for confirmation (Invitrogen, Shanghai, China).

### Analyses of GmFILa protein characteristics and duplication pattern of *YABBY*s

Isoelectric points, protein molecular weights and other protein physicochemical properties were estimated using the ProtParam tool (http://web.expasy.org/protparam/) on the ExPASy proteomics server (http://expasy.org/). Tandem duplication was analyzed based on the method that the distance of two adjacent genes on the same chromosome is less than 200 kb [69]; and segmental duplication was predicted through Plant Genome Duplication Database (http://chibba.agtec.uga.edu/duplication/).

### Phylogenetic and structural analyses

The protein sequences (Additional file 2: Table S4) of 17 soybean *YABBY*s and 29 published plant *YABBY* genes were aligned by ClustalW in MEGA Version 6.0 with the default parameters. A neighbor-joining (NJ) phylogenetic tree was constructed with MEGA 6.0 with the bootstrap of 1000 replications. Soybean *YABBY* genes were named based on the previous report [36]. Gene structures were drawn with the help of GSDS (http://gsds.cbi.pku.edu.cn/).

### Digital expression data analysis

RNA sequencing (RNA-Seq) data, downloaded from SoyBase (http://www.soybase.org/soyseq/), was mainly used to identify the tissue expression of *GmYABBY*s. Soybean microarray expression data, downloaded from Plant Expression Database (http://www.plexdb.org) [70], was specially utilized to analyze the stress response expression patterns of *GmFILa* and other *GmYABBY*s.

### RNA extraction and gene expression analysis

Total RNA was extracted using Plant RNA Extract Kit (TianGen, Beijing, China) according to the manufacturer’s instructions and cDNA was synthetized with M-MLV reverse transcriptase (TaKaRa, Dalian, China). Quantitative real-time polymerase chain reaction (qRT-PCR) was carried out with ABI 7500 system (Applied Biosystems, Foster City, CA, USA) using ChamQ™ SYBR qPCR Master Mix (Vazyme, Nanjing, China). The PCR was performed with the following parameters: 94 °C for 1 min and 40 cycles of 95 °C for 15 s, 60 °C for 15 s, 72 °C for 45 s followed by a final extension at 72 °C for 10 min. The relative expression levels of *GmFILa* were normalized using soybean endogenous gene *tubulin* (GenBank accession no. AY907703) and were estimated utilizing the 2^-ΔΔCt^ method [71]. Semi-quantitative RT-PCR (sqPCR) was conducted with 2 × Hieff™ PCR Master Mix (Yeasen, Shanghai, China), and *Arabidopsis tubulin* gene (AT5G62690) was chosen as an internal control. The PCR protocol was 95 °C for 5 min and 30 cycles of 94 °C for 30 s, 56 °C for 40 s, 72 °C for 1 min followed by a final extension at 72 °C for 10 min. All the gene-specific primer pairs were listed in Additional file 1: Tables S2 and S3.

### Subcellular localization assay of GmFILa protein

The full length of *GmFILa* coding region without a stop codon was inserted into the pBI121-GFP vector to produce the construct 35S:GmFILa-GFP. Both recombinant construct and empty vector 35S:GFP (control) were transferred to onion epidermal cells via particle bombardment method. Laser confocal microscopy (Leica TCS SP2, Mannheim, Germany) was used for image observation.

### mRNA in situ hybridization

Sample (leaf and flower tissues from soybean Nannong 94–16) preparations and mRNA in situ hybridization were performed as previously described [72]. RNA antisense and sense probes were obtained from a 138 bp fragment of the 3′ region of the *GmFILa* cDNA labeled with digoxigenin.

### *Cis*-acting elements in the *GmFILa* promoter region

A 2000 bp fragment upstream the ATG start codon of *GmFILa* was used to evaluate the *cis*-acting elements based on PLACE database (http://www.dna.affrc.go.jp/PLACE/) and Plant CARE (http://bioinformatics.psb.ugent.be/webtools/plantcare/html/).

### Drought and hormone treatments

Soybean Williams82 cultivar was used for drought and hormone treatment experiments. Seeds growth condition and different treatments methods were conducted according to our previous study [73].

### Ectopic expression in *Arabidopsis*

The *GmFILa* CDS was PCR-amplified and introduced into the pBI121 vector. This recombinant construct was transformed into *Arabidopsis* using *Agrobacterium*-mediated transformation following the floral dip method [74]. Transgenic plants were then screened on solid Murashige and Skoog (MS) medium containing 50 μg/ml kanamycin (Kana). Resistant seedlings were transferred to soil and further verified by PCR and sqPCR.

### *Arabidopsis* leaf epidermal cells observation

The 25-day-old leaves of *GmFILa* transgenic and wild type *Arabidopsis* plants were stained with FM4–64 with concentration of 25 μg/ml. After 3 h of dyeing, the plant leaves were observed under confocal microscope (Leica TCS SP2, Mannheim, Germany).

### Leaf paraffin section

The leaves of 25-day-old seedlings were collected from WT and transgenic plants, fixed in FAA (5% formalin, 5% glacial acetic acid and 90% ethanol) at room temperature for more than 24 h, and dehydrated via a graded ethanol series. Further, the samples were embedded in paraffin, sectioned at 6 μm (Leica, RM2135), and stained with safranin. Finally, the stained sections were observed and photographed with light microscope (Leica DMLB).

### Microarray analysis

Leaves of wild *Arabidopsis* and *GmFILa* transgenic plants were sampled for RNA extraction using the tri-reagent (Invitrogen, Gaithersburg, MD, USA). RNA was cleaned using the NucleoSpin® RNA clean-up kit (MACHEREY-NAGEL, Germany), and RNA quality and quantity were assessed with ultraviolet spectrophotometer (NanoDrop Technologies, ND-1000) and formaldehyde agarose gel electrophoresis. Affymetrix *Arabidopsis* Gene Expression Microarray Chip was used for total mRNA hybridization, which was performed by CapitalBio Technology. Fragmentation, hybridization, and washing were carried out using the Hybridization, Wash, and Stain Kit (Affymetrix Technologies) according to the manufacturer’s protocol. Subsequently, the arrays were scanned using GeneChip® Scanner 3000, and images signals (.JPG format) were converted to the digital signals with AGCC software (Affymetrix®GeneChip® Command Console® Software). The data normalization was achieved by using RMA algorithm. Significantly differentially expressed genes (DEGs) between WT and transgenic plants were selected according to these criteria: (a) |Fold Change| (FC) ≥ 2, FC represents expression fold change between wild and transgenic plants; (b) the *P*-value < 0.05; (c) three biological replicates were performed.

### Gene ontology (GO) and Kyoto encyclopedia of genes and genomes (KEGG) enrichment analysis

All the significant DEGs were mapped to GO database using agriGO tool (http://bioinfo.cau.edu.cn/agriGO/analysis.php). The significantly enriched GO terms were evaluated based on P-value (< 0.05). KOBAS 2.0 program (http://kobas.cbi.pku.edu.cn) was used to identify the significantly enriched pathways (P-value < 0.05) in differently expressed genes compared with genome background.

## Additional files

Additional file 1: **Figure S1.** Chromosomal distribution and duplication of soybean *YABBY* genes. **Figure S2.** *GmYABBY*s gene structure analysis. **Figure S3.** Digital tissue expression profiles for soybean *YABBY* genes. **Figure S4.** Expression of eight soybean *YABBY* genes in different tissues/organs based on Plant Expression Database. **Figure S5.** PCR amplification of *GmFILa* CDS from soybean leaf. **Figure S6.** Identification of *GmFILa* transgenic *Arabidopsis* plants. **Figure S7.** Drought tolerance examination of *GmFILa* transgenic and wild type *Arabidopsis* plants. **Table S1.** Duplication analysis of the 17 soybean *YABBY* genes. **Table S2.** Primer pairs of *GmFILa* used for experiments. **Table S3.** Primer pairs of *Arabidopsis* genes used for experiments. (PDF 3710 kb)

Additional file 2: **Table S4.** Protein sequences of *YABBY* genes in various plants. (XLSX 14 kb)

Additional file 3: **Table S5.** Annotation of 82 DEGs between WT and *GmFILa* transgenic *Arabidopsis* plants. (XLSX 21 kb)

Additional file 4: **Table S6.** The 93 GO terms associated with 82 genes. (XLSX 40 kb)

Additional file 5: **Table S7.** The 25 KEGG pathways associated with 41 genes. (XLSX 39 kb)

Additional file 6: **Table S8.** The microarray data (RMA normalized). (XLSX 1398 kb)

## Abbreviations

ABA: Abscisic acid
AM: Apical meristem
CaMV: Cauliflower mosaic virus
CDS: Coding sequence
Col-0: Columbia-0
DAF: Day after flowering
DEG: Differentially expressed genes
GFP: Green fluorescent protein
GO: Gene ontology
h: Hour
IAA: Indole acetic acid
Kana: Kanamycin
KEGG: Kyoto encyclopedia of genes and genomes
MS: Murashige and Skoog
NJ: Neighbor-joining
PEG: Polyethylene glycol
PI: Isoelectric point
qRT-PCR: Quantitative real-time polymerase chain reaction
RNA-seq: RNA sequencing
RT-PCR: Reverse transcription PCR
SA: Salicylic acid
SAM: Shoot apical meristem
sqPCR: Semi-quantitative RT-PCR
WT: Wild type
