# Supplementary material for: Overexpression of a soybean YABBY gene, GmFILa, causes leaf curling in Arabidopsis thaliana
Source: BMC Plant Biol. 2019 Jun 3;19:234. doi: 10.1186/s12870-019-1810-2 (PMC6547562; doi:10.1186/s12870-019-1810-2)
Supplement: Supplementary file 1 — Figure S1. Chromosomal distribution and duplication of soybean YABBY genes. Figure S2. GmYABBYs gene structure analysis. Figure S3. Digital tissue expression profiles for soybean YABBY genes. Figure S4. Expression of eight soybean YABBY genes in different tissues/organs based on Plant Expression Database. Figure S5. PCR amplification of GmFILa CDS from soybean leaf. Figure S6. Identification of GmFILa transgenic Arabidopsis plants. Figure S7. Drought tolerance examination of GmFILa transgenic and wild type Arabidopsis plants. Table S1. Duplication analysis of the 17 soybean YABBY genes. Table S2. Primer pairs of GmFILa used for experiments. Table S3. Primer pairs of Arabidopsis genes used for experiments. (PDF 3710 kb) [file 12870_2019_1810_MOESM1_ESM.pdf]

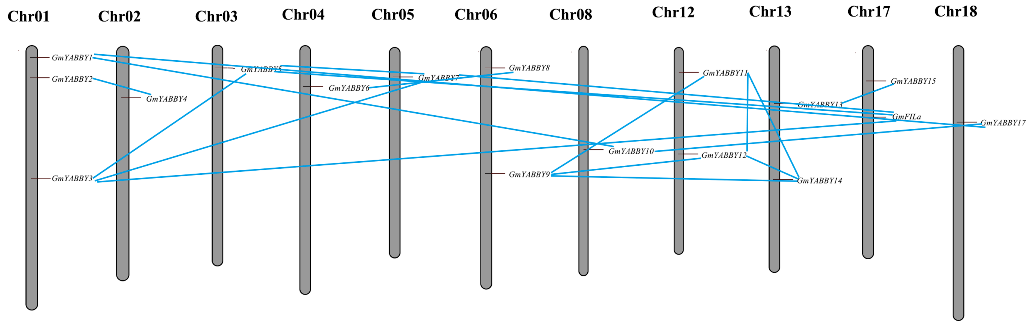

Figure S1. Chromosomal distribution and duplication of soybean *YABBY* genes. *GmYABBY* genes were distributed on the chromosomes with MapDraw. The grey bars represent the chromosomes and the chromosome number is indicated above each bar. Gene locations are shown from top to bottom on the corresponding chromosomes according to soybean genome annotation a2.v1. Segmental duplications are indicated by blue lines.

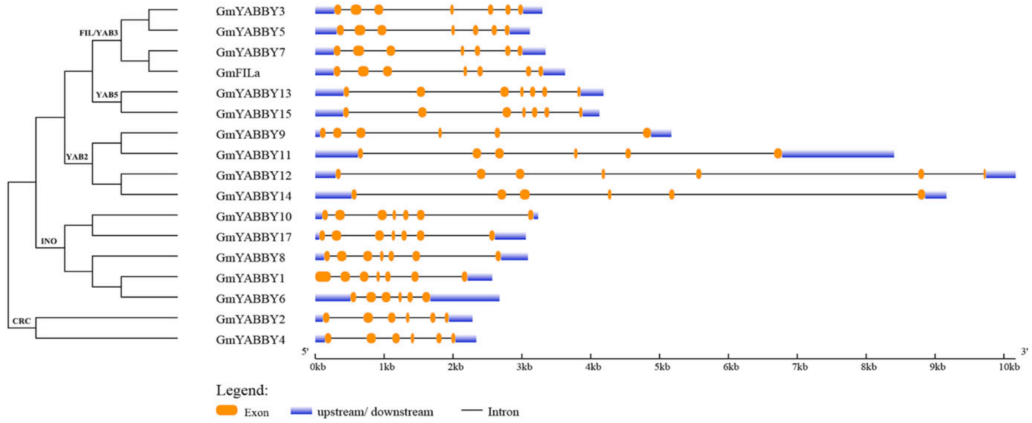

Figure S2. *GmYABBYs* gene structure analysis. The phylogenetic tree and gene structure were constructed using GSDS online website. Exons and introns are designated by orange boxes and black lines, respectively.

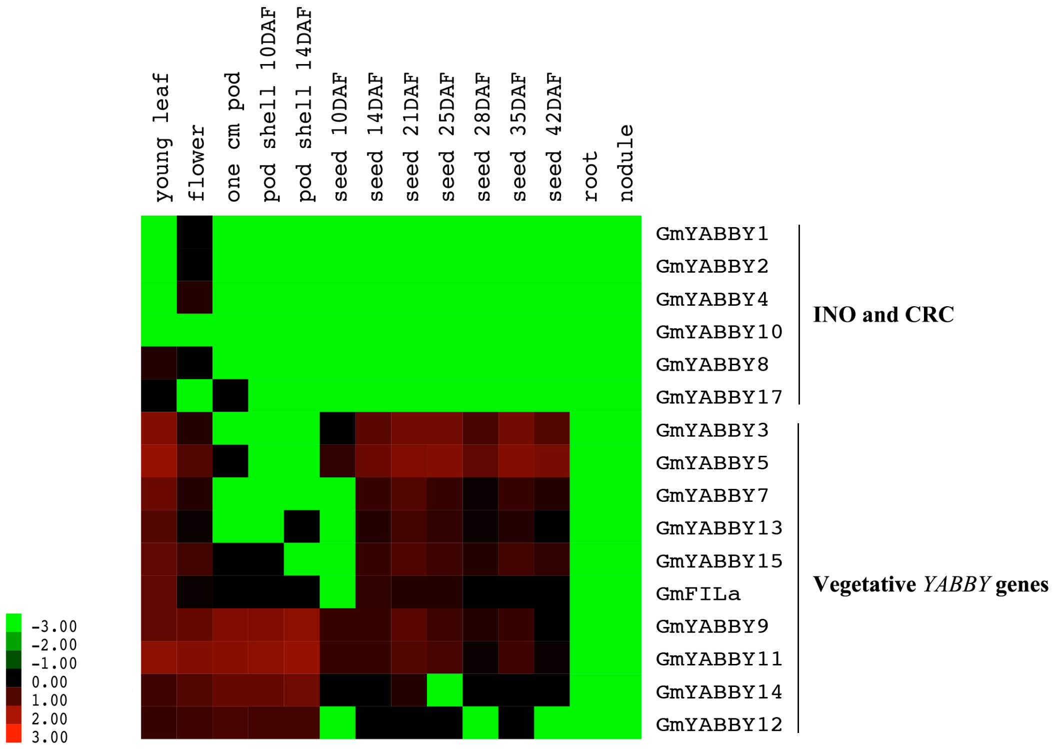

Figure S3. Digital tissue expression profiles for soybean *YABBY* genes. Expression levels of *GmYABBY*s in various tissues based on RNA-seq data from the SoyBase (http://www.soybase.org/). The Reads/Kb/Million (RPKM)-normalized values were log_{10} transformed, and if RPKM=0, log_{10}RPKM=-3. The heat map was drawn with Cluster 3.0 and Java TreeView 1.1.6 software.

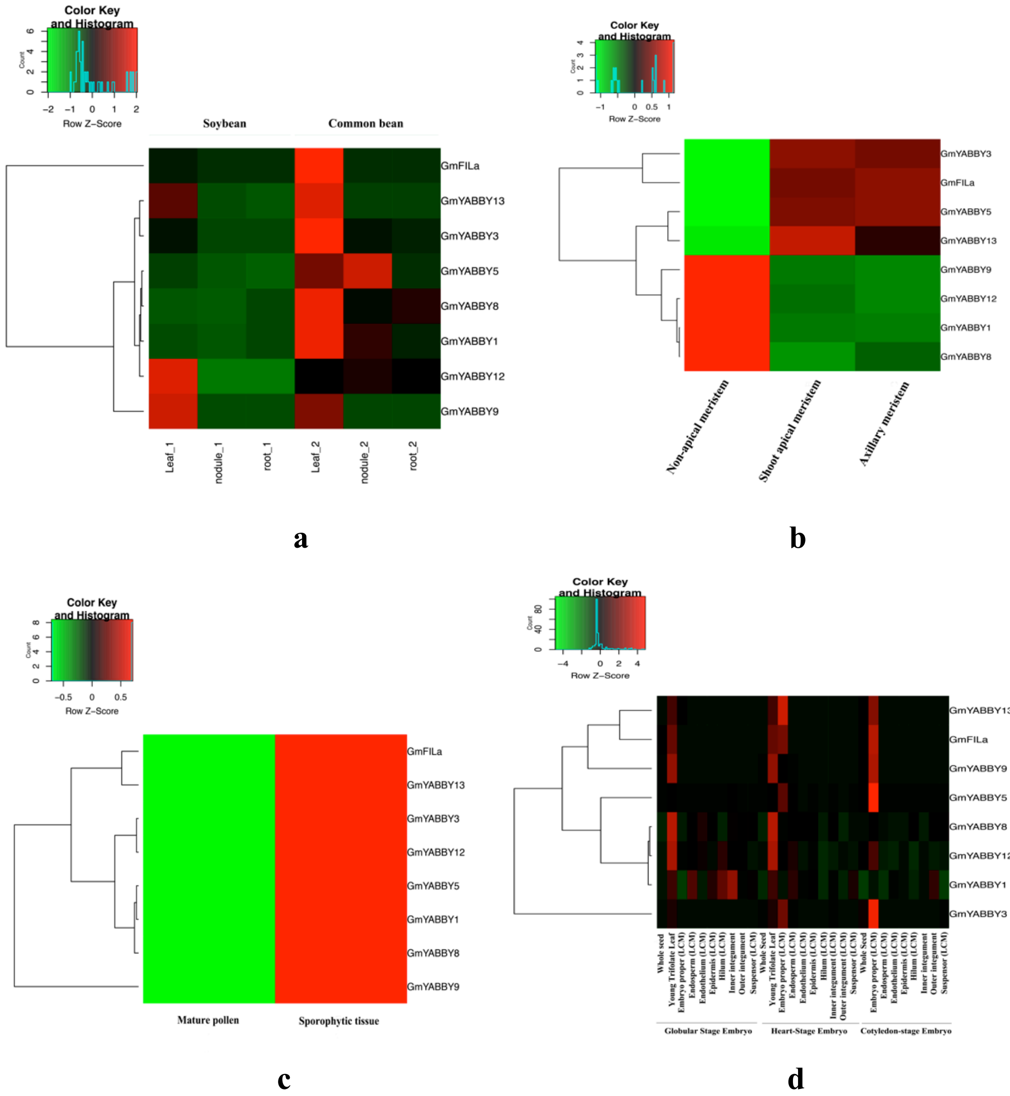

Figure S4. Expression of eight soybean *YABBY* genes in different tissues/organs based on Plant Expression Database. The heat maps were created with MAS-normalized values via local R script.

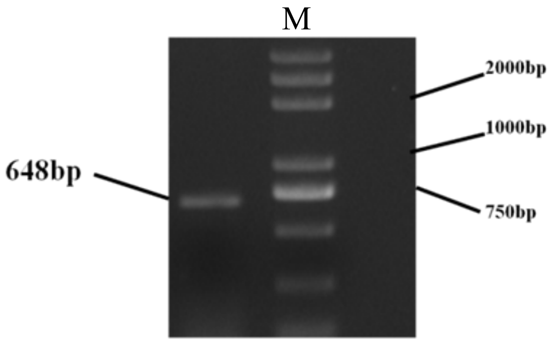

Figure S5. PCR amplification of *GmFILa* CDS from soybean leaf. M: DL 5000 Marker.

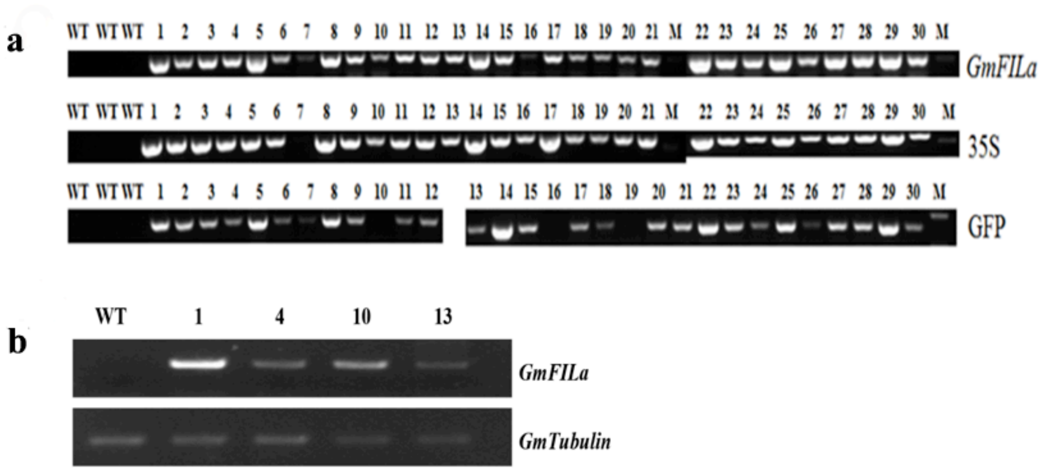

Figure S6. Identification of *GmFILa* transgenic *Arabidopsis* plants. (a) Identification of *GmFILa* transgenic *Arabidopsis* plants by amplifying *GmFILa*, *35s* and *GFP* on genomic DNA, respectively. (b) Semi-quantitative RT-PCR analysis of *GmFILa* expression in wild-type (WT) and 35S:GmFILa lines. WT: Col-0 ecotype. M: Marker. Numerical code: *GmFILa* transgenic lines.

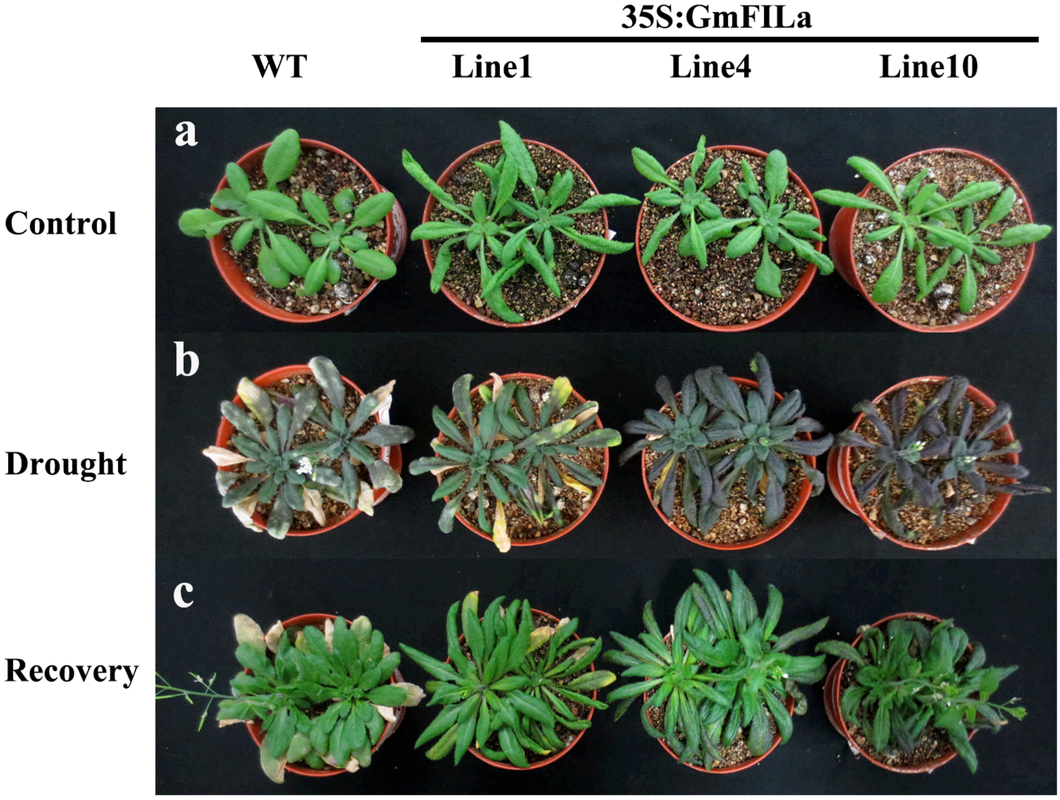

Figure S7. Drought tolerance examination of *GmFILa* transgenic and wild type *Arabidopsis* plants. 25-day-old plants were treated without watering for 2 weeks, and then watered again.

Table S1 Duplication analysis of the 17 soybean *YABBY* genes

Segmental duplication models								
Two-gene		Three-gene			Four-gene			
YABBY2	YABBY4	YABBY1	YABBY10	YABBY17	YABBY3	YABBY5	YABBY7	YABBY16
YABBY6	YABBY8				YABBY9	YABBY11	YABBY12	YABBY14
YABBY13	YABBY15							

Two-gene model indicates a pair of segmentally duplicated genes. Three-gene model represents a segmental duplication involving in three genes. Four-gene model includes four genes, any two of which are paired in a duplicated block.

Table S2 Primer pairs of *GmFILa* used for experiments

Gene Names	Sense primers (5'-3')	Antisense primers (5'-3')	Function
GmFILa	ATCAGATCAGCCTCTTTCCCTC	CCCCAAGAGCTAGTCCTAGTGT	cDNA isolation
GmFILa	TGCTCTAGAATGTCATCCTCTTCCAGC	TCCCCCGGGTTAGTAGGGTGAGACACC	Transformation
GmFILa	TGCTCTAGAATGTCATCCTCTTCCAGC	TCCCCCGGGGTAGGGTGAGACACCAAC	Subcellular localization
GmFILa	TGCTTCGGCTAATGTTGGTGT	TAGGTTGGTGGAACTTGGAAA	qRT-PCR
GmTubulin	GGAGTTCACAGAGGCAGA	CACTTACGCATCACATAGCA	qRT-PCR
GmFILa	CAGATCAGCCTCTTTCCCTC	TGGACAGCTTAACCACCACA	RNA in situ hybridization

Table S3 Primer pairs of *Arabidopsis* genes used for experiments

Gene Names	Sense primers (5'-3')	Antisense primers (5'-3')	Function
35S	AGAGGCTTACGCAGCAGGTC	GCCAGTCTTTACGGCGAGTT	PCR
GFP	AGAGGGTGAAGGTGATGC	TTGTTTGTCTGCCGTGAT	PCR
AtTubulin/ AT5G62690	CTCAAGAGGTTCTCAGCAGTA	TCACCTTCTTCATCCGCAGTT	qRT-PCR/sqPCR
At4g39950	TGTCGATTTCCGGTTCATGA	ACCGTCAGGTGCAGTGTTCTT	qRT-PCR
At1g06160	GTTGAAGAATCTCTCCGGAAGATG	TAGGACGGTTTCTCATGGAGTGT	qRT-PCR
At5g13360	ACTAGCGAGCCTTATCGAGCAA	TGTAACAATGGCTTCGATGCA	qRT-PCR
At5g61600	ACCAAGCGGCGTTTCAATTA	CCGATAACTCCTTCCCCAGAA	qRT-PCR
At2g17500	AGAACCGTCGGGCTCTCTTAC	CTTTCATTGCGGACCCTTTAAT	qRT-PCR
At1g02220	GAGCCATGTCGATCGATTCA	CCAAGCCACATCTTTCAGTTTG	qRT-PCR
At4g22620	TGTGCGGAGGAATGAAACC	CCGGATTCACCGACATGAAC	qRT-PCR
At1g43160	TGTCCTTGGAGAGGCCAAAA	CATACACGTGTCGCCTTGTGT	qRT-PCR
At5g67450	GGCCTCTTCTCCGGTATCGT	TGATGACCGTGATCAAAACGTT	qRT-PCR
